# *Chlamydia pecorum* Ovine Abortion: Associations between Maternal Infection and Perinatal Mortality

**DOI:** 10.3390/pathogens10111367

**Published:** 2021-10-22

**Authors:** Cheryl Jenkins, Martina Jelocnik, Emily Onizawa, Justine McNally, Ronald Coilparampil, Pedro Pinczowski, Daniel Bogema, Thomas Westermann

**Affiliations:** 1 Elizabeth Macarthur Agricultural Institute, NSW Department of Primary Industries, Menangle, NSW 2568, Australia; emily.onizawa@dpi.nsw.gov.au (E.O.); ronald.coilparampil@dpi.nsw.gov.au (R.C.); pedro.pinczowski@dpi.nsw.gov.au (P.P.); daniel.bogema@dpi.nsw.gov.au (D.B.); tww59@cornell.edu (T.W.); 2 Genecology Research Centre, University of the Sunshine Coast, Sippy Downs, QLD 4557, Australia; mjelocni@usc.edu.au; 3 North West Local Land Services, Moree, NSW 2400, Australia; justine.mcnally@lls.nsw.gov.au; 4 Department of Biomedical Sciences, College of Veterinary Medicine, Cornell University, Ithaca, NY 14850, USA

**Keywords:** *Chlamydia pecorum*, abortion, ovine, serology, ST23, plasmid, polymorphic membrane protein

## Abstract

*Chlamydia pecorum* is a common gastrointestinal inhabitant of livestock but infections can manifest in a broad array of clinical presentations and in a range of host species. While *C. pecorum* is a known cause of ovine abortion, clinical cases have only recently been described in detail. Here, the prevalence and sequence types (STs) of *C. pecorum* in ewes from a property experiencing high levels of perinatal mortality (PNM) in New South Wales (NSW), Australia, were investigated using serological and molecular methods. Ewes that were PNM+ were statistically more likely to test seropositive compared to PNM− ewes and displayed higher antibody titres; however, an increase in chlamydial shedding from either the rectum, vagina or conjunctiva of PNM+ ewes was not observed. Multilocus sequence typing (MLST) indicated that *C. pecorum* ST23 was the major ST shed by ewes in the flock, was the only ST identified from the vaginal site, and was the same ST detected within aborted foetal tissues. Whole genome sequencing of *C. pecorum* isolated from one abortion case revealed that the *C. pecorum* plasmid (pCpec) contained a unique deletion in coding sequence 1 (CDS1) that was also present in *C. pecorum* ST23 shed from the ewes. A further unique deletion was noted in a polymorphic membrane protein gene (pmpG) of the *C. pecorum* chromosome, which warrants further investigation given the role of PmpG in host cell adherence and tissue tropism.This study describes novel infection parameters in a sheep flock experiencing *C. pecorum*-associated perinatal mortality, provides the first genomic data from an abortigenic *C. pecorum* strain, and raises questions about possible links between unique genetic features of this strain and *C. pecorum* abortion.

## 1. Introduction

*Chlamydia pecorum* is a globally distributed pathogen infecting a broad range of livestock and wildlife species [1,2,3]. Spillover of infection from domesticated animals into wildlife species, such as the severely impacted Australian marsupial, the koala (*Phascolarctos cinereus*), has been proposed [4]; however, with over 20 animal species now recognised as harbouring this organism [5], cross-transmission pathways are likely to be extremely complex.

Infection with *C. pecorum* can be subclinical or manifest as a range of clinical syndromes including polyarthritis, keratoconjunctivitis, pneumonia, enteritis, and encephalomyelitis, as well as reproductive losses through infertility and abortion [6]. In livestock hosts, these syndromes impose a substantial burden on production with sheep, cattle, goats and pigs all susceptible to *C. pecorum* infection. Infections that are clinically inapparent also have the potential to impact production, with one study demonstrating a 48% reduction in the growth rate of calves [7]. Effects on the growth rate of sheep have not yet been demonstrated, although longitudinal studies have shown that a high proportion of Australian lambs (31%) shed *C. pecorum* subclinically in their faeces [8]. Studies conducted in Europe have demonstrated similar rates of infection in clinically healthy sheep flocks of between 5 and 50% [9,10]. Clinical ovine infections commonly manifest as polyarthritis or keratoconjunctivitis. While cases of abortion associated with *C. pecorum* are also known to occur and have been demonstrated under both experimental [11] and natural [12] conditions, they are less well defined. Indeed, *C. abortus* represents the major abortigenic *Chlamydia* species globally [10,13] except for Australia and New Zealand, where this species is considered to be exotic [6].

Despite the wide range of hosts infected by *C. pecorum* and the array of diseases caused by this organism, to date, genomic analyses are limited to a total of 17 sheep, koala, and pig strains, all of which display greater than 98% sequence similarity. Single nucleotide polymorphisms (SNPs) within the *Chlamydia* plasticity zone and genes encoding the polymorphic membrane proteins (PMPs) account for most of the inter-strain differences [14]. These genomic analyses also identified that the majority of livestock and koala *C. pecorum* strains are plasmid-bearing; however, plasmidless strains are also common across all hosts. Multilocus sequence typing (MLST) has revealed associations between sequence type (ST) 23 and multiple disease presentations in sheep and cattle [4], however vaginorectal and asymptomatic enteric infections with genetically diverse STs are also common in these hosts. 

We recently described the pathological features associated with naturally occurring *C. pecorum* ovine abortion on a farm in New South Wales (NSW), Australia [12] and confirmed that ST23 is correlated with abortion cases. Here, we used serological and quantitative PCR (qPCR) testing to determine the prevalence of *C. pecorum* infection in ewes within the same flock, used MLST to investigate the links between maternal infection and perinatal mortality (PNM) caused by *C. pecorum* ST23, and undertook whole genome sequencing to further characterise the strain involved in the abortion cases.

## 2. Results

### 2.1. Prevalence of C. pecorum

**Figure 1:**
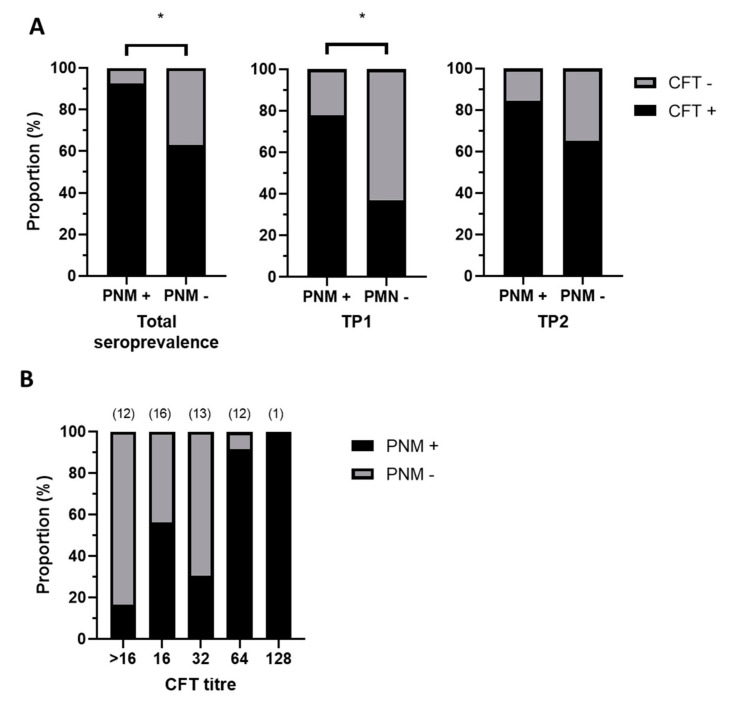
(**A**) Total *Chlamydia* seroprevalence in ewes (left panel) was significantly higher (*) in PNM+ compared to PNM− ewes (*p* = 0.019); however, this effect was only significant at TP1 (*p* = 0.005; middle panel), not TP2 (*p* = 0.263; right panel). (**B**) The majority of ewes with *Chlamydia* CFT titres > 32 were PNM+. Numbers of ewes with CFT titres < 16, 16, 32, 64 and 128 are displayed in brackets above the bars.

The *C. pecorum* seroprevalence amongst the ewes included in this study (n = 54) was 77.78% (Supplementary Table S1). There was evidence of a statistically significant association (Fisher exact test: *p* = 0.019) between seropositivity and PNM, with PNM+ ewes having approximately 7 × greater odds of testing positive for *C. pecorum* using CFT (OR = 7.35; 95% CI = 1.536–35.61). When sampling time point was accounted for, this observation was significant at TP1 (Fisher exact test: OR = 5.95; 95% CI = 1.826–17.81; *p* = 0.005), but not at TP2 (Fisher exact test: OR = 2.96; 95% CI 0.56–15.90; *p* = 0.263) (Figure 1A). This likely reflects exposure of a proportion of ewes to *C. pecorum* post-lambing, as a significantly higher proportion of ewes were seropositive at TP2 compared to TP1 (McNemar test: *p* = 0.01). High CFT titres (>32) were almost always associated with PNM (Figure 1B and Supplementary Table S1).

**Figure 2:**
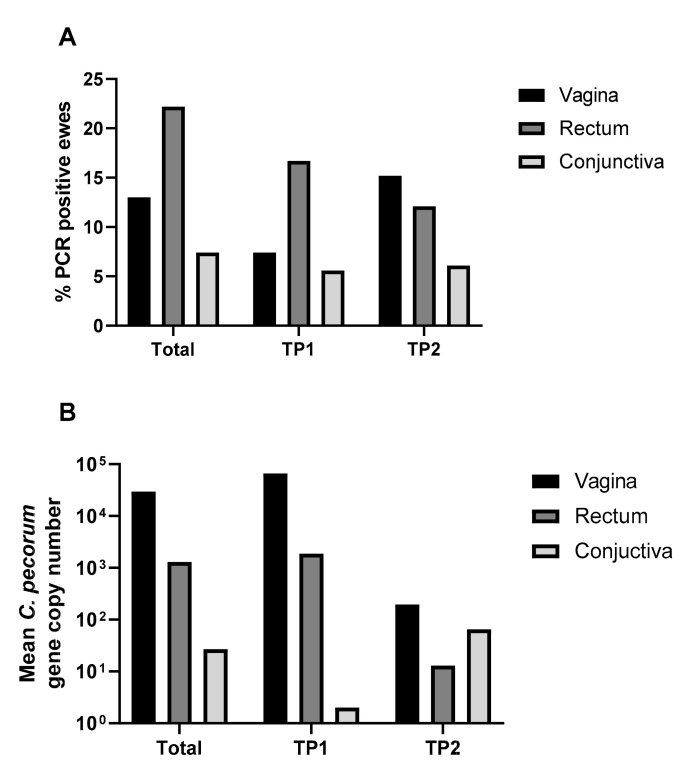
Proportion of ewes shedding *C. pecorum* from rectal, vaginal and conjunctival sites (**A**) and mean gene copy number shed from each site (**B**) Over the entire study period and at TP1, the majority of ewes shed *C. pecorum* from the rectal site (**A**), but shedding was heaviest from the vaginal site (**B**).

Positive *Chlamydia* genus and *C. pecorum* qPCR results correlated with each other, with the latter qPCR assay used to calculate gene copy numbers. All samples tested negative for *C. abortus*. We detected *C. pecorum* by qPCR in a total of 17/54 ewes from at least one anatomical site and one sampling timepoint, indicating active shedding in 31.5% of the ewes over the study period (Figure 2A, Supplementary Table S1). There was no statistical association (*p* = 0.766) between the prevalence of *C. pecorum* shedding and perinatal mortality (Fisher exact test: OR = 0.70; 95% CI = 0.219–2.155). *C. pecorum* shedding was detected most often from the rectal site however the load of *C. pecorum* detected at the vaginal site was higher on average than from either the rectum or the conjunctiva (Figure 2B). There was no statistical difference in the number of ewes shedding *C. pecorum* at TP1 compared to TP2 (McNemar test: *p* = 0.68). 

There was no association between seropositivity and chlamydial shedding at either timepoint (Fisher exact test: *p* = 0.80).

### 2.2. Whole Genome Analysis of Ovine Abortion Strain 18-13680-18FL

In our previous study describing the gross and microscopic lesions associated with *C. pecorum* abortion, we confirmed that the *C. pecorum* ST involved in the three abortion cases examined on the NSW property was ST23 [14]. In this study, we further investigated the abortigenic strain 18-13680-18FL cultured from a foetal lung from one of those cases using whole genome sequencing on DNA extracts from tissue culture supernatant. The assembly generated from *C. pecorum* 18-13680-18FL grown in McCoy B cells (murine origin) showed a total of 1683 contigs. These were primarily identified as fragmented host *Mus musculus* sequences with a small number (n = 46) also identified belonging to *Mycoplasma orale*. Two sequences matched *C. pecorum* in BLASTN searches, with one contig similar in size and sequence to the *C. pecorum* chromosome and another matching in size and sequence to the previously characterised plasmid, pCpec [14]. No other contigs matched other *Chlamydia* spp. in BLASTN searches. Assembly statistics for the 18-13680-18FL draft genome before and after extraction of *Chlamydia* contigs are shown in Supplementary Table S2 and a blobplot (generated by blobtools) showing sequence coverage vs. %GC for each BLASTN match is shown in Supplementary Figure S1. Attempts to circularise the putative 18-13680-18FL chromosome failed due to a long repeat region located adjacent to the 5S rRNA subunit gene, however alignments with the *C. pecorum* E58 chromosome showed that the 18-13680-18FL is not missing shared gene content at the edges of the sequence (data not shown). 

**Figure 3:**
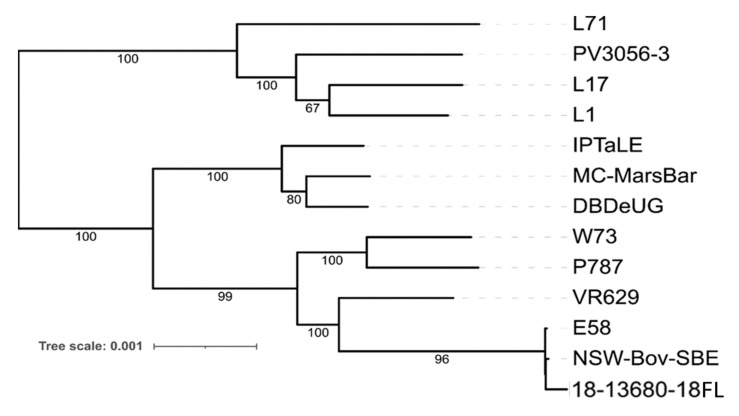
Maximum likelihood core-genome phylogenetic tree of *C. pecorum* sequences from this study compared to reference sequences. RefSeq sequences are labelled with strain identifier. Branch support values are listed adjacent to the calculated branch.

Phylogenetic analysis of the 18-13680-18FL core genome and existing *C. pecorum* reference sequences indicated that 18-13680FL is most closely related to two strains isolated from cattle: the type strain, E58, and NSW Bov SBE (Figure 3). Both strains were isolated from cases of sporadic bovine encephalomyelitis (SBE).

We also examined the pairwise core genome SNP distance between 18-13680-18FL and complete *C. pecorum* genomes in found in RefSeq. 18-13680-18FL was most closely related to strains E58 and NSW-Bov-SBE, with 236 and 240 core genome SNPs, respectively (Supplementary Table S3). Other complete genomes showed core SNP differences of between 3000 and 7000 with the closest sheep strain being VR629 (IPA) (3171 SNPs), which was isolated from a polyarthritis case. The 18-13680-18FL genome displayed the greatest number of SNPs (>6000) with the three porcine strains (L1, L17 and L71) and a strain isolated from a bovine metritis case (PV3056-3).

**Figure 4:**
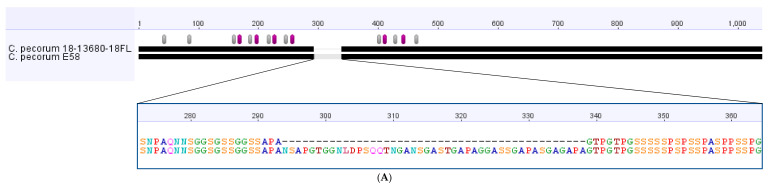

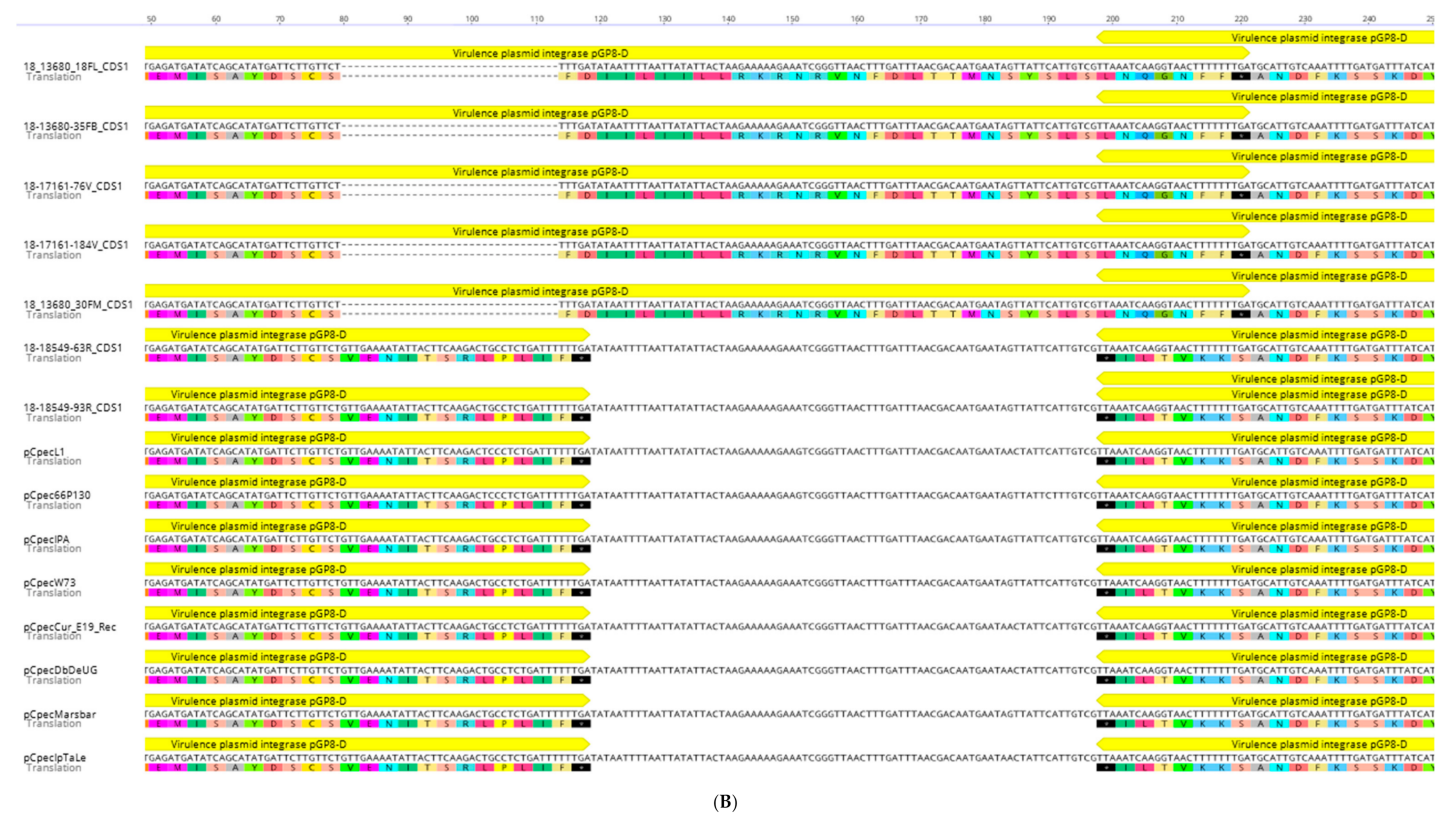

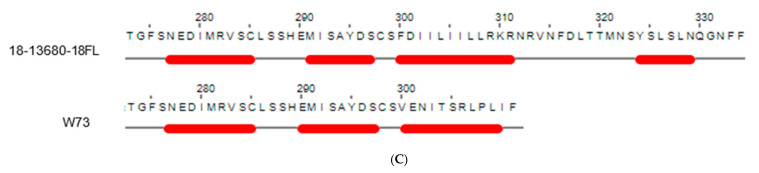
(**A**) Amino acid alignment of the polymorphic membrane protein G (PmpG). *C. pecorum* strain 18-13680-18FL contains a 45 aa (135 bp) deletion in the N-terminal portion of PmpG which contains numerous tetrapeptide repeats of sequence GGAI (purple bars) and FxxN (grey bars) that are important for adherence to host cells. The deletion occurs in a region of low complexity within the N-terminal region of PmpG. (**B**) Nucleotide alignment of the 3′ end of CDS1 of pCpec with the translated protein sequences shown. The strain associated with ovine abortion (18-13680-18FL) contains a 34 bp deletion in CDS1 (encoding plasmid integrase). The nucleotide deletion results in deletion of 11 amino acids and causes a frameshift mutation which removes the stop codon allowing readthrough and elongation of the protein by 34 amino acids. The 18-13680-18FL CDS1 protein is therefore predicted to be 23 amino acids longer than the W73 CDS1 protein. (**C**) Secondary structure predictions indicate that the C-terminal alpha helix of W73 CDS1 protein is preserved in 18-13680-18FL which also contains an additional C-terminal tail. Alpha helices are indicated by the red bars.

In addition to the individual SNPs identified, strain 18-13680-18FL also displayed a 135bp in-frame deletion in the pmpG. This deletion was confirmed via Sanger sequencing and was not observed in pmpG from any other *C. pecorum* strains with sequenced genomes, including the closely related E58 and NSW/Bov/SBE strains (Figure 4A). The deletion is in the tetrapeptide (GGAI and FxxN)-containing N-terminal portion of the PMP in a region of low-complexity rich in serine, glycine and proline (Figure 4A). 

The *C. pecorum* plasmid (pCpec) from 18-13680-18FL shared between 98.68 and 99.42% sequence identity with pCpec plasmids from reference *C. pecorum* strains. In contrast, pCpec from the reference strains all share 99.10–99.99% sequence identity with each other [15]. The lower % sequence identity observed between 18-13680-18FL and the reference strains was largely due to the presence of a previously uncharacterised 34 bp deletion in CDS1 of 18-13680-18FL pCpec, representing a 3′ segment of the plasmid integrase gene (Figure 4B). If this deletion was disregarded, 18-13680-18FL pCpec displayed >99% identity with pCpec from all of the reference strains and was most similar to pCpec from strain W73 (99.87%) which was cultured from sheep faeces [16]. The 34 bp deletion in CDS1 results in an 11-amino acid deletion and a frameshift mutation in the predicted protein sequence. The frameshift mutation removes the stop codon and is predicted to result in readthrough of the protein and the addition of 34 amino acids (Figure 4B). The resultant protein is therefore predicted to be 23 amino acids longer than the usual product of CDS1. Comparison of the predicted secondary structures of the 18-13680-18FL and W73 CDS1 proteins indicate that the C-terminal alpha helix of the W73 CDS1 protein is preserved in 18-13680-18FL despite the shift in the primary amino acid sequence; however, a region rich in basic amino acids (RKRNR) and an additional alpha helix is predicted to occur in the C-terminal tail of the 18-13680-18FL CDS1 protein (Figure 4C).

### 2.3. MLST of C. pecorum from Vaginal, Rectal and Conjunctival Sites

Samples from the sheep flock that tested positive in qPCR screening for *C. pecorum* were subjected to MLST to determine whether the abortigenic strain (ST23) could be linked to strains being shed from the vaginal, rectal or conjunctival sites. A total of 11 samples including rectal swabs (n = 5), vaginal swabs (n = 5) and a conjunctival swab (n = 1) contained sufficient *C. pecorum* DNA to enable successful MLST amplification. Results of the MLST are shown in Table 1 and Supplementary Figure S2. ST23 was identified in 8/11 samples tested and across all three body sites. ST23 was the only sequence type identified in vaginal swabs but ST206 was also identified in rectal swabs along with two novel STs (305 and 307) (Supplementary Table S4). The ST23 strains from this study clustered in a well-supported clonal clade with other Australian and global ST23, detected in disease cases (ST metadata shown in Supplementary Table S5). The rectal STs (206, 305 and 307) clustered in a genetically diverse clade with other mostly rectal STs, forming their own lineages (Supplementary Figure S2). The ST206 detected in sample 18-18549-105R was identical to that of strain P787, isolated from a joint in a sheep polyarthritis case from Scotland. 

As the pCpec plasmid was detected in the genome sequence derived from the cultured foetal lung sample (18-13860-18FL), we screened rectal, vaginal and conjunctival samples from the ewes as well as the remaining foetal samples for the presence of pCpec. pCpec was detected in all samples tested with the exception of two vaginal swabs (Table 1). Gene copy calculations suggested that the plasmid copy number was 1:1 relative to the *C. pecorum* chromosome (data not shown). When samples containing the pCpec plasmid were screened by PCR and sequencing for the deletion in the 3′ end of plasmid integrase gene (CDS1), all samples harbouring ST23 were found to contain the CDS1 deletion while samples with ST206, 305 and 307 were not (Table 1). 

## 3. Discussion 

While *C. pecorum* has historically been reported in association with abortion in small ruminants [17,18], the associated fetoplacental lesions have only recently been described in detail [12,19,20], and knowledge of the epidemiology of *C. pecorum*-induced abortion is lacking. In this study we investigated the prevalence and sequence types of *C. pecorum* present in a sheep flock that had been identified as experiencing *C. pecorum*-induced perinatal mortality. The high *Chlamydia* seroprevalence identified in the flock over the study period, (approx. 78%) suggested that a large proportion of the flock was exposed to *C. pecorum*. While seropositivity was significantly higher amongst PNM+ ewes earlier in the study period, a large proportion of PNM−ewes also tested seropositive. This is consistent with prior studies of *C. pecorum*-induced arthritis in sheep that demonstrated that seropositivity at the flock level is a more reliable indicator of infection at the flock level rather than at an individual animal level [21]; however, in this study high CFT titres (>32) were almost always associated with PNM. The lack of correlation between high CFT titres and chlamydial shedding was not unexpected based on observations of prior studies of *C. pecorum* infections in sheep [21,22] with an estimated lag time between exposure and seroconversion of 1–2 months [22].

At 31.5%, the prevalence of shedding of *C. pecorum* over the study period (as measured by qPCR) was similar to that observed in prior studies on sheep in Australia and Europe which have demonstrated anywhere from 15–60% prevalence in the absence of high rates of abortion [8,21,23,24]. Therefore, the prevalence of *C. pecorum* shedding observed in this study was unremarkable despite high rates of PNM. Given that the gastrointestinal tract is the major niche occupied by *C. pecorum*, frequent shedding from the rectal site was expected, although the load of *C. pecorum* was higher on average from the vaginal site. Whether this is reflective of clinical infection of the reproductive tract is unclear as the small sample number of ewes with vaginal shedding did not allow for meaningful comparsions between those with and without PNM. A prior longitudinal study on clinically normal lambs indicated that the load of *C. pecorum* shed from the vagina was age-dependent, increasing after 4 months and remaining so until the study endpoint at 10 months. The possibility of similar increases in shedding occurring at 2–4 months after initial vagina infection, regardless of age, is also considered [22]. A limitation of this study was the sampling of ewes 4–5 weeks after abortions were noted on farm; earlier sampling may have enabled increased detection of ewes with vaginal chlamydial shedding.

Previously, we demonstrated that three abortion cases from this NSW property were caused by *C. pecorum* ST23 [12]. Here, we demonstrated that ST23 was also the dominant sequence type shed from both PNM+ and PNM− ewes within the affected sheep flock. ST23 is considered to be a virulent sequence type that has been associated with a range of clinical presentations in both sheep and cattle [24] and was also recently linked to a report of ovine abortion in Western Australia [20]. Pathogenic ST23 is genetically distinct from less virulent sequence types that are associated with the gastrointestinal tract and shed in the faeces [4,24]; however, in this study we identified ST23 in each body site, including the rectal site. ST23 was the only sequence type identified in vaginal swabs and the single conjunctival swab tested, as well as in tissues from aborted lamb foetuses [12]. A greater diversity of STs was identified in rectal swabs with three additional STs being identified. Of these additional STs, only ST206 has been identified previously in Europe in the synovial fluid of a sheep with polyarthritis (strain P787) [16], while ST305 and ST307 both represent novel STs. Nevertheless, all three STs cluster with other previously described rectal and/or faecal strains.

Strain 18-13680-18FL, which was isolated from the lung of one of the aborted lamb foetuses in our prior study [12], was subjected to whole genome sequencing to determine how this strain compared to *C. pecorum* ST23 isolated from other hosts and clinical disease presentations. To the best of our knowledge, the newly assembled genome is the first genomic characterisarion of an abortigenic *C. pecorum*. While both phylogenetic and SNP analysis of the core genome indicated that 18-13680-18FL was highly similar to two strains associated with SBE from cattle, some unique features were identified. A 135 bp deletion in the pmpG gene of strain 18-13680-18FL was unique amongst *C. pecorum* strains for which genome sequences were available. The Pmp proteins form part of the outer membrane complex of *Chlamydia* spp. along with the genus-specific lipopolysaccharide (LPS) and outer membrane proteins [25], and have been shown to be important for host cell adhesion in *C. trachomatis* [26], *C. pneumoniae* [27] and *C. abortus* [28]. In *C. trachomatis*, pmp genes cluster phylogenetically based on tissue of origin [29] and have been shown to determine both tissue tropism and species specificity [26]. Pmp proteins are highly immunogenic in murine models [30] and in sheep following enzootic abortion [31]. For these reasons, Pmp proteins have frequently been included in chlamydial vaccine formulations [28,32,33,34], including *C. pecorum* [35]. The pmp gene regions are a major source of diversity amongst *C. pecorum* genomes; however, the majority of SNPs within this region result in synonymous substitutions [14]. It was notable, therefore, that the pmpG gene of 18-13680-18FL contained a large, in-frame deletion. This deletion is located within the N-terminal portion of PmpG which contains numerous tetrapeptide repeats and has been shown to be important in host cell interactions and the formation of adhesion-competent multimeric filaments [27,36]. The deleted region did not result in a decrease in the number of tetrapeptide repeats relative to the type strain E58, but occurred in a low complexity region between repeats that is rich in serine, proline and glycine. Recent research suggests that low complexity regions in proteins perform important functions in bacteria such as polysaccharide receptor activity [37]. The presence of this deletion is novel within *C. pecorum* PmpGs examined so far and further investigation is warranted to determine if it is associated with enhanced virulence of this strain and/or correlated with tropism for the placenta. It would be of particular interest to determine whether this deletion is present in the abortigenic strain of *C. pecorum* recently identified in Western Australia [20] as well as other cases detected in NSW [38].

Plasmids are widespread and highly conserved amongst members of the *Chlamydia* genus and therefore are believed to play an important role in virulence. The greatest diversity in plasmid sequence is observed amongst *C. pecorum* strains, in which 12 different genotypes (denoted A-L) of the plasmid, pCpec, have been identified [15]. pCpec from the abortigenic strain 18-13680-18FL characterised in this study is of a unique genotype but is most closely related to Genotype G, previously identified in strain W73 isolated from sheep faeces [15]. The 18-13680-18FL pCpec differed from W73 pCpec by a number of individual SNPs and the presence of a 34 bp deletion in CDS1, which encodes the plasmid integrase gene. This deletion is unique amongst pCpec sequences previously examined; however, a deletion in the CDS1 gene of pCpnE1 from C. pneumoniae strain N16 [39] and the plasmids of two strains of C. trachomatis (Swedish-nvCT and Mexican-nvCT) has been reported [40,41,42]. In the case of Swedish-nvCT, this deletion resulted in failure of molecular diagnostic tests targeting this region and the subsequent spread of chlamydia cases caused by the deletion-containing variant. This demonstrates that deletions in CDS1 do not necessarily result in reduced virulence [43]. While the deletion in CDS1 of 18-13680-FL pCpec is predicted to result in the removal of a stretch of 11 conserved amino acids, interestingly this deletion also results in a frameshift mutation that removes the stop codon, thereby allowing readthrough of the protein for a further 34 amino acids. Frameshift mutations resulting in protein elongation are much less common than mutations that result in protein truncation and can enhance, alter, or be deleterious to protein function [44]. It was notable that the alpha helical structure created by the deleted 11 C-terminal amino acids of the CDS1 protein is predicted to be replicated by the inserted amino acids; however, the functional implications of the additional 23 amino acids at the C-terminus of the 18-13680-18FL CDS1 protein require further investigation. The significance of the novel deletion in pCpec 18-13680-18FL is unclear and it was not present in all *C. pecorum* strains associated with PNM; however, it is notable that this deletion was only associated with ST23 in the current study. Given that both the deletion in CDS1 of pCpec and the deletion in the pmpG gene are unique amongst *C. pecorum* strains, they could serve as useful markers for strain tracking.

## 4. Materials and Methods

### 4.1. Farm History and Sample Collection

The flock examined consisted of 1350 sheep, including pregnant maidens (n = 464), multiparous pregnant ewes (n = 600), and non-pregnant animals (n = 286). A low rate of lambing was observed in the maiden ewes (66%) relative to the multiparous ewes (136%) and compared to the pregnancy rates (100% and 159%, respectively) as determined by ultrasound scanning. Stillbirths and abortions were observed amongst the maiden ewes between September and October of 2018 and testing of three aborted foetuses from the maiden group confirmed that *C. pecorum* was the likely cause [12]. 

To further investigate the epidemiology of the abortions on farm, a subset of 54 ewes from the affected farm were sampled over a one-week period in late October/early November of 2018 (Time point 1; TP 1) including those experiencing perinatal mortality (PNM+; n = 27) and those that lambed successfully (PNM−; n = 27). Follow up sampling was conducted 4-5 weeks later on the 7th of December (Time point 2; TP2) on 32 available ewes from the PNM+ (n = 12) and PNM− (n = 20) groups.

All samples were collected by a Local District veterinarian as part of a clinical and diagnostic investigation, which precluded any requirement for specific animal ethics approval. Samples collected included venous jugular blood for serological testing and rectal, vaginal, and conjunctival swabs for molecular testing. Swabs were cotton-tipped dry swabs and they were placed into phosphate buffered gelatine saline (PBGS) for transport to the laboratory.

### 4.2. Serological Testing

Sheep were tested for exposure to *C. pecorum* using the complement fixation test (CFT). The CFT detects antibodies to *Chlamydia* genus-specific antigens and can be used to detect all chlamydial infections. CFT testing was carried out in 96 well ‘U’ bottom microtiter plates (Greiner Bio-One, Kremsmünster, Austria). A doubling dilution of sera in Kolmer diluent (150 mM NaCl; 0.2 mM CaCl_2_; 0.63 mM MgCl_2_; 0.2 mM sodium azide) were tested following inactivation by heating at 60 °C for 45 min in an incubator. The inactivated and diluted sera were then mixed with *Chlamydia pecorum* antigen (elementary bodies) and guinea pig complement diluted to 1:50 and 1:60, respectively, in Kolmer diluent and incubated at 37 °C for 1 h. Twenty five microlitres of haemolysin-sensitised 3% sheep red blood cells (RBCs) were then added to the serum/antigen/complement mixture and incubated at 37 °C for 30 min on a plate shaker (Titramax 1000, Heidolph, Schwabach, Germany) at 1000–1200 rpm. Following incubation, plates were centrifuged at 524× *g* and the results read on a light box. Inhibition of haemolysis demonstrated the presence of specific antibody while complete haemolysis indicated the absence of detectable levels of specific complement fixing antibodies. The reciprocal of the highest dilution of serum showing ≤50% (2+) haemolysis indicated the antibody titre of the serum. Reactions of 2+ or greater at a dilution ≥ 1:16 were considered positive.

### 4.3. Quantitative PCR (qPCR)

DNA was extracted from transport medium containing vaginal, rectal and conjunctival swabs for molecular testing. Five hundred microlitres of PBGS medium was pelleted at 13,000 rpm in a benchtop microfuge and DNA extracted using Instagene matrix (Bio-Rad, Hercules, CA, USA) according to the manufacturer’s instructions. The qPCRs for Chlamydia genus and *C. pecorum* were carried out as described previously in [12] using primers designed by Ehricht et al. [45] and Pantchev et al. [46], respectively. Positive and negative controls were included in each run and plasmid standards diluted in tRNA [12] were run in duplicate in each assay to enable calculation of the number of gene copies of *C. pecorum* in each sample. *C. abortus* was also excluded as a possible cause of infection using a specific qPCR for this species as previously described [12,46].

### 4.4. Statistical Analysis

All calculations were performed in GraphPad (Prism, version 8.2.0). To assess whether there was an association between seropositivity and perinatal mortality, a Fisher exact test was used with no association being the null hypothesis. Changes between the two time points in the proportion of CFT or *C. pecorum* qPCR positive results were tested using McNemar’s test.

### 4.5. Multilocus Sequence Typing (MLST)

To evaluate genotypes of infecting strains, MLST was performed on *C. pecorum* qPCR positive extracts from vaginal, rectal and conjunctival swabs as outlined by Jelocnik et al. [4]. This typing scheme targets seven different housekeeping genes (gatA, oppA_3, hflX, gidA, enoA, hemN and fumC). Upon Sanger sequencing, resulting chromatograms and sequences were analysed in Geneious Prime 2021.1.1 (https://www.geneious.com, accessed on 30 July 2021). Sequence types were assigned using the Chlamydiales typing database hosted at PubMLST: https://pubmlst.org/, (accessed on 30 July 2021) [47] and novel sequence types were also deposited into this database. The concatenated MLST sequences from this study were aligned using ClustalOmega (as implemented in Geneious Prime 2021.1.1) to 40 additional global and Australian publicly available livestock *C. pecorum* ST sequences retrieved from the Chlamydiales PubMLST database. Using the 3059 bp alignment of a total of 51 concatenated *C. pecorum* MLST sequences, a mid-point rooted approximately-maximum-likelihood phylogenetic tree was constructed using FastTree 2.1.11 with GTR + G model for nucleotide substitutions [48] (as implemented in Geneious Prime 2021.1.1). The metadata for each ST were displayed using Phandango [49].

### 4.6. Whole Genome Sequencing (WGS)

To further characterise the strain involved in the abortion cases, C. pecorum from a previous abortion case that had been cultured in McCoy cells in [12] was subjected to whole genome sequencing using an Ion Torrent S5. DNA was extracted from tissue culture supernatant using the KingFisher Flex purification system (ThermoFisher, Waltham, MA, USA) and eluted in molecular grade water prior to quantification on a Qubit Fluorometer (Thermofisher). A library was prepared from 100 ng of genomic DNA by fragmentation of the sample using the Ion Xpress^TM^ Plus Fragment Library Kit (Thermofisher catalog no. 4471269) and incubating for 7 min at 37 °C before magnetic bead purification using ethanol washes. DNA was eluted in low TE buffer. DNA was ligated and barcodes added at 25 °C for 15 min followed by 72 °C for 5 min. The library was purified again using the magnetic bead method and products of approximately 450 bp in length were recovered using the E-gel precast system. Amplification of the library was achieved using the following conditions: 1 × cycle of 95 °C for 5 min followed by 9 × cycles of 95 °C for 15 s; 58 °C for 15 s and 70 °C for 60 s. The amplified library was purified and diluted 1:100 before real-time PCR quantification on a ViiA 7 against Escherichia coli standards using the Ion Universal Library Quantitation kit (ThermoFisher catalog no. A26217). The cycling conditions for the PCR were as follows: 50 °C for 2 min; 95 °C for 20 s followed by 40 × cycles of 95 °C for 1 s and 60 °C for 20 s. The quantified library was diluted to a concentration of 100 pM and loaded onto an Ion 520 chip using the Ion Chef system. The prepared chip was then run on the Ion Torrent S5 next generation sequencing system. To ensure adequate coverage of the C. pecorum assembly two libraries were generated from one sample of purified DNA using the above method.

### 4.7. Genome Assembly and Analysis

Basecalled sequencing output from two Ion-Torrent runs were merged then assembled and quality-checked using a previously established pipeline (https://github.com/bogemad/asqcan, accessed on 1 March 2020). Briefly, read quality was established with FastQC [50]. Reads were assembled with SPAdes v3.13.0 [51] utilising the --careful and --iontorrent options. Assembly QC metrics were calculated with QUAST v5.0.2 [52]. Identification of *Chlamydia* spp. sequences was achieved using blobtools v1.01 [53] and BLASTN [54] (options: -max_target_seqs 10 -max_hsps 1 -evalue 1e-25) with the NCBI nt database (accessed 28 March 2019). *Chlamydia* spp. contigs were extracted from metagenomic assemblies using a custom python script (https://gist.github.com/bogemad/84488444f6d776fbd08defb64db445de, accessed on 1 March 2020) and blobtools/blastn results. Small indels associated with homopolymer repeats on the Ion Torrent platform were corrected manually by alignment to the *C. pecorum* E58 chromomosome and pCpec from strain W73 [55]. Genome annotation was achieved with Prokka v1.13.3 using default options [56]. Sequencing reads from both libraries were deposited in SRA under accession numbers SRR15184364 and SRR15184365. The 18-13680-18FL genome assembly was deposited in GenBank under the accession number CP9079918.

For phylogenetic and pairwise SNP analysis, selected *C. pecorum* assemblies (n = 12) were collected from NCBI RefSeq. A core genome alignment was generated using Snippy v4.3.6 and snippy-core with alignment columns containing gaps and ambiguous bases trimmed. Core genome SNP counts were calculated directly from alignments using biopython [57]. Phylogeny was inferred from the core-genome alignment using IQ-TREE (http://www.iqtree.org, accessed on 20 June 2021) with the ModelFinder algorithm used to select the optimal base substitution model [58]. Branch support values were estimated using the ultrafast bootstrap (UFBoot) method within IQ-TREE, utilising 1000 replications [59]. The inferred tree was rooted with FastRoot using minimum variance [60].

### 4.8. Detection of the C. pecorum Plasmid

Extracts of *C. pecorum* positive conjunctival, vaginal and rectal swabs were tested for the presence of the *C. pecorum* plasmid (pCpec) using the conventional PCR and qPCR screening assays previously described in [14] and [61] targeting a 522 bp conserved intergenic region and 233 bp fragment of coding domain sequence 5 (CDS5) of pCpec, respectively. In order to screen pCpec-containing samples for the 34 bp pCpec CDS1 deletion identified in 18-13680-18FL by WGS, we used previously described forward 5′ CCACACGTATTACGAGCTA 3′ and reverse 5′ CGTCTTGGATGTTCGGACTC 3′ primers [15] to generate a 944 bp amplicon spanning the deleted region. The conventional PCR reactions were performed in 35 µL volume, consisting of 17.5 µL Amplitaq Gold mix (ThermoFisher, Scoresby, Australia), 12.5 µL PCR grade water, 1 µL of each 10 µM forward and reverse primer (total concentration at 0.3 µM) and 3 µL DNA template. The cycling conditions were as follows: initial denaturation at 95 °C for 10 min, followed by 35 cycles of 95 °C for 20 s, 52.5 °C for 45 s, 72 °C for 45 s and a final extension at 72 °C for 7 min. Positive (plasmid-bearing strain *C. pecorum* Marsbar DNA) and negative (MilliQ water) controls were included in each assay. PCR products were electrophoresed on a 1.5% agarose gel, followed by visual confirmation under a UV transilluminator. Based on band intensity and DNA concentration, amplicons were bidirectionally Sanger sequenced at Macrogen (Seoul, South Korea). Chromatograms and sequence analyses were also performed in Geneious Prime 2021.1.1 (https://www.geneious.com, accessed on 30 July 2021). Predictions of the secondary structure of the C-terminal region of the CDS1 protein were made with JPred (https://www.compbio.dundee.ac.uk/jpred/, accessed on 15 September 2021).

## 5. Conclusions

This study describes infection parameters in a sheep flock experiencing *C. pecorum*-associated abortions. High *Chlamydia* antibody titres in ewes were strongly associated with perinatal mortality, while chlamydial shedding, as measured by qPCR, was not. We also provided the first genomic characterisation of an abortigenic strain of *C. pecorum* and identified novel deletions in the pCpec plasmid and the pmpG gene. Future studies should focus on genetic profiling of other globally distributed abortigenic strains of *C. pecorum* to determine whether novel genetic characteristics observed in this study are important for tissue tropism. The molecular characterisation of further abortigenic *C. pecorum* strains may aid in future vaccine development for this important livestock pathogen.

## Figures and Tables

**Table 1:** Results of MLST and presence of pCpec and the pCpec CDS1 deletion in samples collected from rectal, vaginal and conjunctival swabs from perinatal mortality (PNM)+ and PNM− ewes.

Sample Name	Host (ID)	Sample Type	PNM	ST	Reference	pCpec	CDS1 Deletion
18-18549-63R	Ewe (7)	Rectal swab	+	307 *	This study	+	−
18-17161-75R	Ewe (24)	Rectal swab	+	23	This study	+	+
18-18549-93R	Ewe (36)	Rectal swab	+	305 *	This study	+	−
18-18549-105R	Ewe (48)	Rectal swab	+	206	This study	+	−
18-17161-147R	Ewe (25)	Rectal swab	−	23	This study	+	+
18-17161-76V	Ewe (24)	Vaginal swab	+	23	This study	+	+
18-18549-115V	Ewe (4)	Vaginal swab	+	23	This study	−	N/A
18-18549-137V	Ewe (25)	Vaginal swab	−	23	This study	−	N/A
18-18549-143V	Ewe (31)	Vaginal swab	−	23	This study	+	+
18-17161-184V	Ewe (31)	Vaginal swab	−	23	This study	+	+
18-18549-190C	Ewe (23)	Conjunctival swab	−	23	This study	+	+
18-13680-29FM	Foetus 1	Foetal Membranes	+	23	[12]	+	+
18-13680-30FM	Foetus 2	Foetal Membranes	+	23	[12]	+	+
18-13680-22FK	Foetus 3	Foetal Kidney	+	23	[12]	+	+
18-13680-35FB	Foetus 3	Foetal Brain	+	23	[12]	+	+
18-13680-18FL	Foetus 1	Foetal Lung Culture	+	23	[12]	+	+

* Denotes novel STs.

## Data Availability

Novel MLST data has been deposited in PubMLST—https://pubmlst.org/, accessed on 30 July 2021. Genome sequence reads were deposited in the Sequence Read Archive (SRA) under accession numbers SRR15184364 and SRR15184365. The 18-13680-18FL genome and plasmid assemblies were deposited in GenBank under the accession numbers CP9079918 and CP079919, respectively.

## Supplementary Materials

The following are available online at https://www.mdpi.com/article/10.3390/pathogens10111367/s1, Figure S1: Blobplot showing sequence coverage vs. %GC for each contig in the raw assembly generated by Spades, Figure S2: Phylogenetic relationships of the *C. pecorum* strains from ewes from this study and other livestock hosts. Table S1: Results of serological testing and qPCR testing of vaginal, rectal and conjunctival swabs from PNM positive and negative ewes, Table S2: 18-13680-18FL assembly statistics before and after extraction of *Chlamydia* spp. contigs, Table S3: SNPs between *C. pecorum* strain 18-13680-18FL sequenced in this study and other *C. pecorum* strains from different hosts, Table S4: Results for individual MLST loci for rectal, vaginal and conjunctival swabs from ewes and foetal tissues from abortion cases, Table S5: Metadata associated with *C. pecorum* STs from this study and presented in Figure S2.
