# Supplementary material for: Chlamydia pecorum Ovine Abortion: Associations between Maternal Infection and Perinatal Mortality
Source: Pathogens. 2021 Oct 22;10(11):1367. doi: 10.3390/pathogens10111367 (PMC8618313; doi:10.3390/pathogens10111367)
Supplement: Supplementary file 1 [file pathogens-10-01367-s001.zip › Figure S1.pdf]

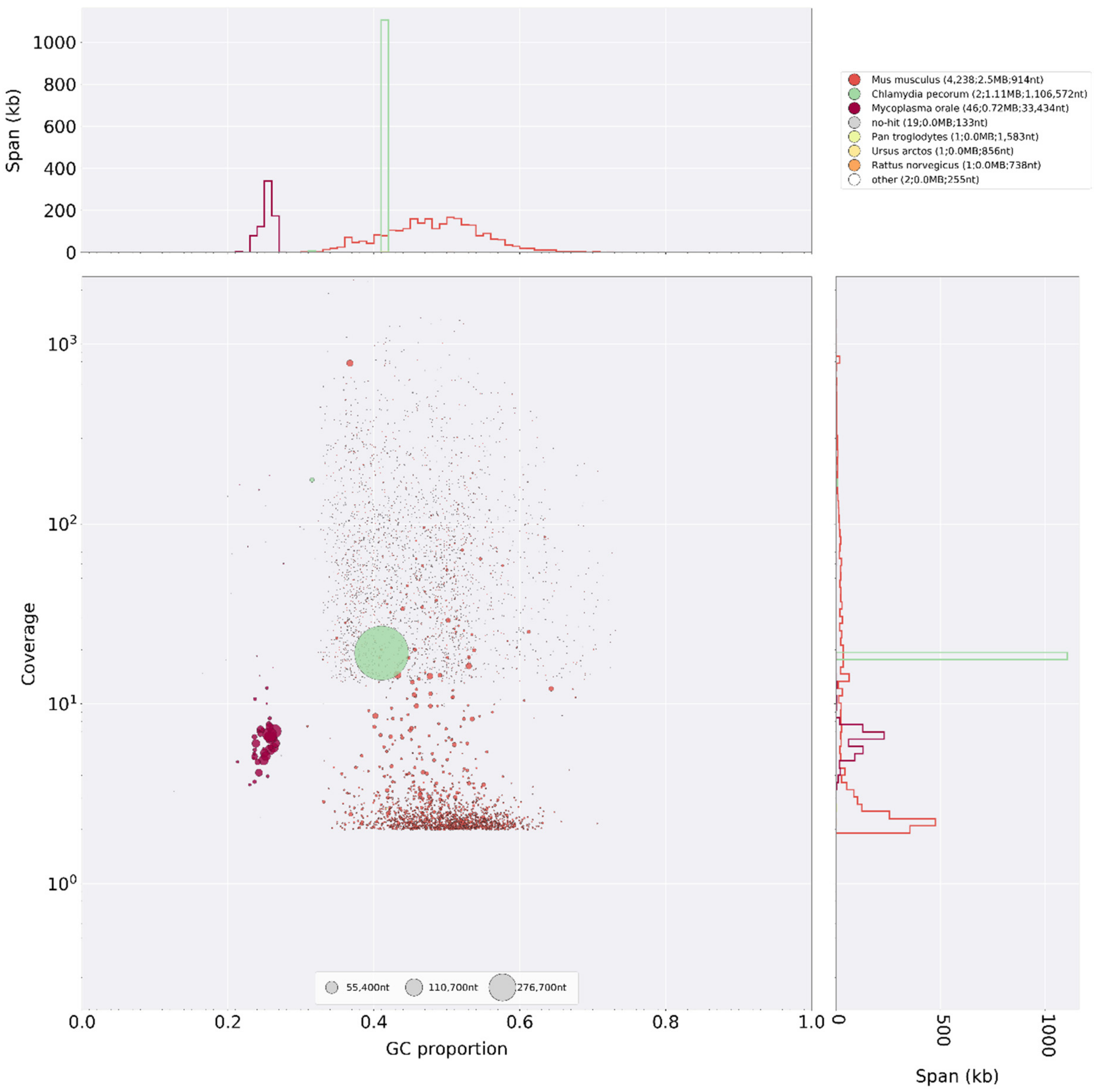

Figure S1. Blobplot showing sequence coverage vs %GC for each contig in the raw assembly generated by Spades. Scaffolds are coloured by best BLASTN match and scaffold size is represented by blob size. Two *C. pecorum* scaffolds were identified by BLASTN and represented the chromosome and plasmid. These scaffolds were separated for further study. Figure was generated with Blobtools.
