# Supplementary material for: Chlamydia pecorum Ovine Abortion: Associations between Maternal Infection and Perinatal Mortality
Source: Pathogens. 2021 Oct 22;10(11):1367. doi: 10.3390/pathogens10111367 (PMC8618313; doi:10.3390/pathogens10111367)
Supplement: Supplementary file 1 [file pathogens-10-01367-s001.zip › Figure S2.pdf]

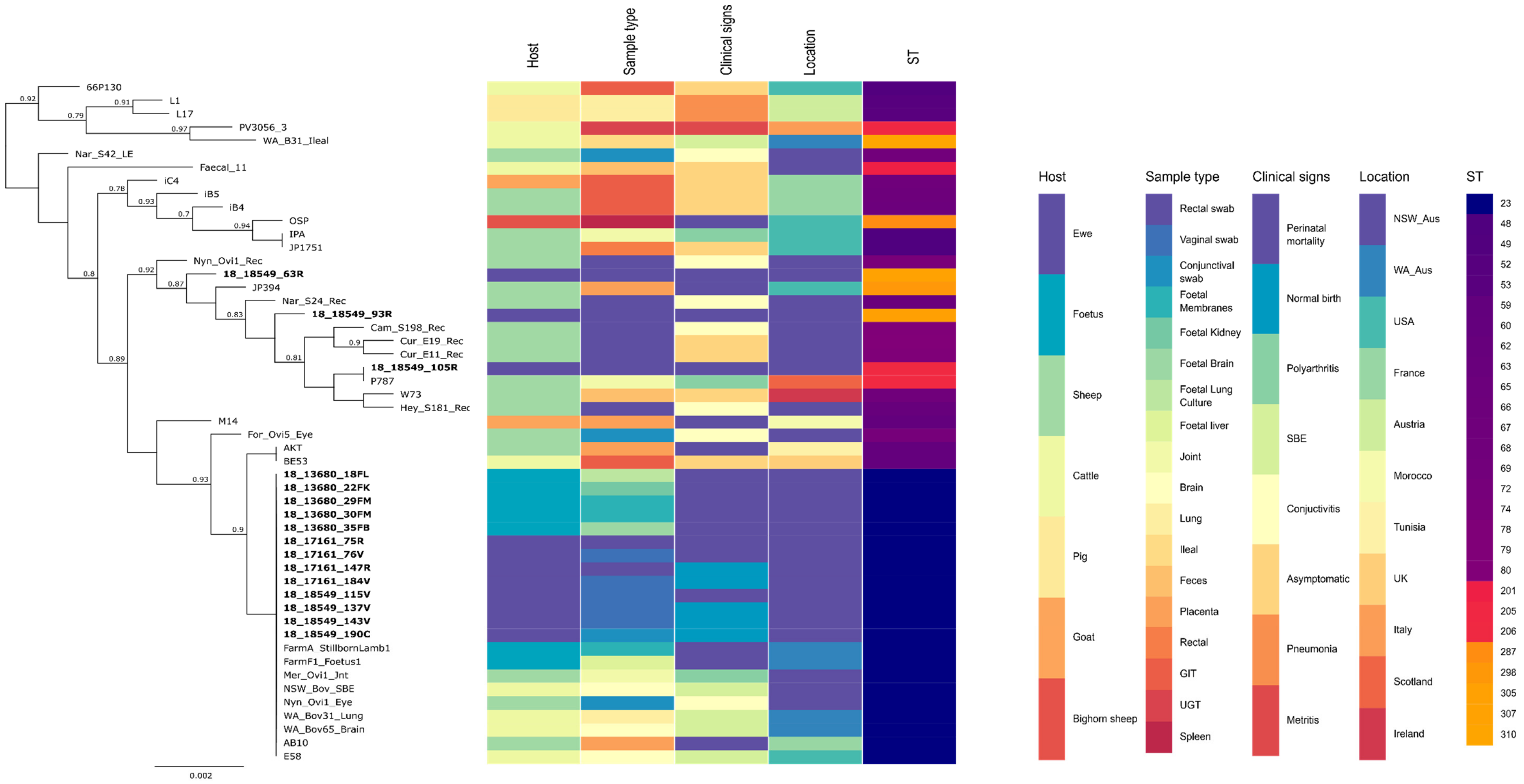

Figure S2. Phylogenetic relationships of the *C. pecorum* strains from ewes from this study and other livestock hosts. The mid-point rooted tree was constructed using a 3095 bp concatenated MLST sequences alignment from the 51 *C. pecorum* strains, including the ewes and foetal *C. pecorum* strains described in this and previous study [14] (highlighted in bold). Support values of > 0.7 are displayed on the tree nodes. Metadata for each strain denotes: hosts where sheep, ewe and foetus are denoted separately for descriptive reasons; sample types; clinical signs; geographical location where for Australia two regions are denoted (Aus_NSW and Aus_WA); and STs.
