# Supplementary material for: Chlamydia pecorum Ovine Abortion: Associations between Maternal Infection and Perinatal Mortality
Source: Pathogens. 2021 Oct 22;10(11):1367. doi: 10.3390/pathogens10111367 (PMC8618313; doi:10.3390/pathogens10111367)
Supplement: Supplementary file 1 [file pathogens-10-01367-s001.zip › Table S1.pdf]

Table S1. Results of serological testing and qPCR testing of vaginal, rectal and conjunctival swabs from PNM positive and negative ewes

Animal ID	PNM	CFT titre TP1	CFT titre[a] TP2	qPCR TP1[b] (swab type)	qPCR TP2[b] (swab type)
1	(+)	<8	16	-	-
2	(+)	<8	16	-	-
3	(-)	<8	64	-	-
4	(+)	128	16	+ V, R, C	+ V
5	(+)	64	32	-	-
6	(+)	32	64	-	-
7	(+)	64	32	+ R	+ R
8	(+)	<8	16	-	-
9	(-)	<8	8	+ C	+ C
10	(-)	16	16	-	-
11	(+)	16	32	-	-
12	(+)	32	32	-	-
13	(+)	<8	<8	-	-
14	(-)	32	32	-	-
15	(-)	8	16	-	-
16	(+)	16	16	-	-
17	(+)	16	8	-	-
18	(-)	<8	8	-	-
19	(-)	8	<8	-	-
20	(-)	<8	16	-	-
21	(-)	8	16	-	-
22	(+)	64	NT	-	-
23	(-)	<8	<8	-	+ C
24	(-)	<8	16	-	+ V, R
25	(-)	<8	32	+ R	+ V
26	(-)	32	16	-	+ V
27	(-)	16	16	-	+ R
28	(-)	<8	8	-	-
29	(-)	<8	32	-	-
30	(-)	<8	8	-	-
31	(-)	<8	8	+ V, R	+ V
32	(-)	16	16	+ R	-
33	(+)	<8	16	+ V	+ R
34	(-)	32	16	+ V	-
35	(-)	<8	NT	-	-
36	(+)	16	NT	+ R	-
37	(-)	32	NT	-	-
38	(+)	16	NT	+ R	-
39	(-)	8	NT	+ C	-
40	(+)	32	NT	-	-
41	(+)	64	NT	-	-
42	(+)	64	NT	-	-

43	(+)	64	NT	-	-
44	(-)	32	NT	-	-
45	(+)	64	NT	-	-
46	(+)	64	NT	-	-
47	(+)	64	NT	+ R	-
48	(+)	64	NT	+ R	-
49	(+)	32	NT	-	-
50	(+)	8	NT	-	-
51	(-)	32	NT	-	-
52	(-)	32	NT	-	-
53	(+)	16	NT	-	-
54	(-)	<8	NT	-	-

[a]NT = not tested

[b]V = vaginal swab, R = rectal swab, C = conjunctival swab
