# Supplementary material for: Chlamydia pecorum Ovine Abortion: Associations between Maternal Infection and Perinatal Mortality
Source: Pathogens. 2021 Oct 22;10(11):1367. doi: 10.3390/pathogens10111367 (PMC8618313; doi:10.3390/pathogens10111367)
Supplement: Supplementary file 1 [file pathogens-10-01367-s001.zip › Table S2.pdf]

Table S2: 18-13680-18FL assembly statistics before and after extraction of *Chlamydia* spp. contigs

18-13680-18FL	Raw assembly - pre-extraction	Raw assembly - Post-extraction	Polished assembly Chromosome	Polished assembly Plasmid
# contigs	1,683	2	1	1
Largest contig	1,106,572	1,106,572	1,105,979	7,513
Total length	3,846,963	1,114,218	1,105,979	7,513
GC (%)	42.17	40.99	41.07	31.55
Coverage depth (×)	18.02	20.24	19.16	176.04
N50	6975	1,106,572	1,105,979	7,513
L50	41	1	1	1
# N's/100 kbp	0	0	0	0
