# Supplementary material for: Chlamydia pecorum Ovine Abortion: Associations between Maternal Infection and Perinatal Mortality
Source: Pathogens. 2021 Oct 22;10(11):1367. doi: 10.3390/pathogens10111367 (PMC8618313; doi:10.3390/pathogens10111367)
Supplement: Supplementary file 1 [file pathogens-10-01367-s001.zip › Table S3.pdf]

Table S3. SNPs between *C. pecorum* strain 18-13680-18FL sequenced in this study and other *C. pecorum* strains from different hosts.

C.pecorum strain (host)	18-13680-18FL	E58	DBDeUG	IPTaLE	L1	L17	L71	MC-MarsBar	NSW-Bov-SBE	P787	PV3056-3	VR629	W73
18-13680-18FL (Ovine)	0	236	4926	4978	6275	6536	6646	4923	240	3979	6567	3171	3917
E58 (Bovine)	236	0	4735	4785	6091	6336	6446	4732	50	3783	6383	2975	3724
DBDeUG (Koala)	4926	4735	0	1558	5444	5710	6354	1220	4749	4261	5742	4041	4196
IPTaLE (Koala)	4978	4785	1558	0	5667	5815	6411	1564	4803	4379	5901	4155	4279
L1 (Porcine)	6275	6091	5444	5667	0	2379	3799	5461	6107	6810	2687	6651	6797
L17 (Porcine)	6536	6336	5710	5815	2379	0	3727	5702	6352	6992	2810	6853	6962
L71 (Porcine)	6646	6446	6354	6411	3799	3727	0	6298	6462	7161	4033	6975	7034
MC-MarsBar (Koala)	4923	4732	1220	1564	5461	5702	6298	0	4748	4365	5860	4057	4256
NSW-Bov-SBE (Bovine)	240	50	4749	4803	6107	6352	6462	4748	0	3801	6399	2993	3740
P787 (Ovine)	3979	3783	4261	4379	6810	6992	7161	4365	3801	0	7091	2887	2063
PV3056-3 (Bovine)	6567	6383	5742	5901	2687	2810	4033	5860	6399	7091	0	6932	7043
VR629 (Ovine)	3171	2975	4041	4155	6651	6853	6975	4057	2993	2887	6932	0	2817
W73 (Ovine)	3917	3724	4196	4279	6797	6962	7034	4256	3740	2063	7043	2817	0
