# Supplementary material for: Chlamydia pecorum Ovine Abortion: Associations between Maternal Infection and Perinatal Mortality
Source: Pathogens. 2021 Oct 22;10(11):1367. doi: 10.3390/pathogens10111367 (PMC8618313; doi:10.3390/pathogens10111367)
Supplement: Supplementary file 1 [file pathogens-10-01367-s001.zip › Table S5.pdf]

Table S5. Metadata associated with *C. pecorum* STs from this study and presented in Figure S2.

Sample name	Host	Sample type	Clinical signs	Location	ST
18_18549_63R	Ewe	Rectal swab	Perinatal mortality	NSW_Aus	307
18_17161_75R	Ewe	Rectal swab	Perinatal mortality	NSW_Aus	23
18_18549_93R	Ewe	Rectal swab	Perinatal mortality	NSW_Aus	305
18_18549_105R	Ewe	Rectal swab	Perinatal mortality	NSW_Aus	206
18_17161_147R	Ewe	Rectal swab	Normal birth	NSW_Aus	23
18_17161_76V	Ewe	Vaginal swab	Perinatal mortality	NSW_Aus	23
18_18549_115V	Ewe	Vaginal swab	Perinatal mortality	NSW_Aus	23
18_18549_137V	Ewe	Vaginal swab	Normal birth	NSW_Aus	23
18_18549_143V	Ewe	Vaginal swab	Normal birth	NSW_Aus	23
18_17161_184V	Ewe	Vaginal swab	Normal birth	NSW_Aus	23
18_18549_190C	Ewe	Conjunctival swab	Normal birth	NSW_Aus	23
18_13680_29FM	Foetus	Foetal Membranes	Perinatal mortality	NSW_Aus	23
18_13680_30FM	Foetus	Foetal Membranes	Perinatal mortality	NSW_Aus	23
18_13680_22FK	Foetus	Foetal Kidney	Perinatal mortality	NSW_Aus	23
18_13680_35FB	Foetus	Foetal Brain	Perinatal mortality	NSW_Aus	23
18_13680_18FL	Foetus	Foetal Lung Culture	Perinatal mortality	NSW_Aus	23
FarmA_StillbornLamb1	Foetus	Foetal Membranes	Perinatal mortality	WA_Aus	23
FarmF1_Foetus1	Foetus	Foetal liver	Perinatal mortality	WA_Aus	23
Mer_Ovi1_Jnt	Sheep	Joint	Polyarthritis	NSW_Aus	23
NSW_Bov_SBE	Cattle	Brain	SBE	NSW_Aus	23
WA_Bov65_Brain	Cattle	Brain	SBE	WA_Aus	23
WA_Bov31_Lung	Cattle	Lung	SBE	WA_Aus	23
WA_B31_Ileal	Cattle	Ileal	SBE	WA_Aus	310
Hey_S181_Rec	Sheep	Rectal swab	Conjunctivitis	NSW_Aus	62
Nar_S24_Rec	Sheep	Rectal swab	Conjunctivitis	NSW_Aus	63
Cur_E11_Rec	Sheep	Rectal swab	Asymptomatic	NSW_Aus	78
Cur_E19_Rec	Sheep	Rectal swab	Asymptomatic	NSW_Aus	80
Faecal_11	Cattle	Feces	Asymptomatic	NSW_Aus	201
For_Ovi5_Eye	Sheep	Conjunctival swab	Conjunctivitis	NSW_Aus	72
Nyn_Ovi1_Eye	Sheep	Conjunctival swab	Conjunctivitis	NSW_Aus	23
Nyn_Ovi1_Rec	Sheep	Rectal swab	Conjunctivitis	NSW_Aus	74
Cam_S198_Rec	Sheep	Rectal swab	Conjunctivitis	NSW_Aus	79
Nar_S42_LE	Sheep	Conjunctival swab	Conjunctivitis	NSW_Aus	69
E58	Cattle	Brain	SBE	USA	23
AB10	Sheep	Placenta	Perinatal mortality	France	23
IPA	Sheep	Joint	Polyarthritis	USA	48
JP1751	Sheep	Rectal	Asymptomatic	USA	48
66P130	Cattle	GIT	Asymptomatic	USA	49
L1	Pig	Lung	Pneumonia	Austria	52
L17	Pig	Lung	Pneumonia	Austria	53
M14	Goat	Placenta	Perinatal mortality	Morocco	59
AKT	Sheep	Placenta	Perinatal mortality	Tunisia	60
BE53	Cattle	GIT	Asymptomatic	UK	60
iB4	Sheep	GIT	Asymptomatic	France	65
iB5	Sheep	GIT	Asymptomatic	France	66

iC4	Goat	GIT	Asymptomatic	France	67
PV3056_3	Cattle	UGT	Metritis	Italy	205
P787	Sheep	Joint	Polyarthritis	Scotland	206
JP394	Sheep	Placenta	Perinatal mortality	USA	298
W73	Sheep	Faeces	Asymptomatic	Ireland	68
OSP	Bighorn sheep	Spleen	Perinatal mortality	USA	287
